# The Cholecystokinin Type 2 Receptor, a Pharmacological Target for Pain Management

**DOI:** 10.3390/ph14111185

**Published:** 2021-11-19

**Authors:** Amandine Bernard, Aurore Danigo, Sylvie Bourthoumieu, Mohamad Mroué, Alexis Desmoulière, Franck Sturtz, Amandine Rovini, Claire Demiot

**Affiliations:** 1 EA6309—MMNP, Faculties of Medicine and Pharmacy, University of Limoges, 87025 Limoges, France; amandine.bernard@unilim.fr (A.B.); sylvie.bourthoumieu@unilim.fr (S.B.); mohamad.mroue@unilim.fr (M.M.); alexis.desmouliere@unilim.fr (A.D.); franck.sturtz@unilim.fr (F.S.); amandine.rovini@unilim.fr (A.R.); claire.demiot@unilim.fr (C.D.); 2 Department of Cytogenetic, Medical Genetic and Reproduction Biology, University Hospital of Limoges, 87025 Limoges, France; 3 Department of Biochemistry and Molecular Genetics, University Hospital of Limoges, 87025 Limoges, France

**Keywords:** cholecystokinin type 2 receptor, pain management, antagonist, rodent model, clinical trial

## Abstract

Over the past decades, accumulating evidence has demonstrated a pivotal role of cholecystokinin type 2 receptor (CCK2R) in pain modulation. The established role of CCK2R activation in directly facilitating nociception has led to the development of several CCK2R antagonists, which have been shown to successfully alleviate pain in several rodent models of pain. However, the outcomes of clinical trials are more modest since they have not demonstrated the expected biological effect obtained in animals. Such discordances of results between preclinical and clinical studies suggest reconsidering our knowledge about the molecular basis of the pharmacology and functioning of CCK2R. This review focuses on the cellular localization of CCK2R specifically in the sensory nervous system and discusses in further detail the molecular mechanisms and signal transduction pathways involved in controlling pain perception. We then provide a comprehensive overview of the most successful compounds targeting CCK2R and report recent advances in pharmacological strategies used to achieve CCK2R modulation. We purposely distinguish between CCK2R benefits obtained in preclinical models and outcomes in clinical trials with different pain etiologies. Lastly, we emphasize the biological and clinical relevance of CCK2R as a promising target for the development of new treatments for pain management.

## 1. Introduction

Chronic pain and its management remains a major health care issue and presents a therapeutic challenge, in large part because of its complex underlying pathophysiology. During the past decade, many studies have provided evidence of a pain modulating role for the G protein-coupled cholecystokinin type 2 receptor, CCK2R. Cholecystokinin (CCK) is a peptide that was discovered in the porcine gastrointestinal tract by Ivy and Oldberg in 1928 [1] and soon became part of the family of classical gut hormones, together with secretin and gastrin. As such, CCK is involved in exocrine [2] and endocrine [3,4] pancreatic secretion, gallbladder contraction and gut motility [5]. Two types of receptors mediate the action of CCK: cholecystokinin receptor type 1 CCK1R, formerly known as CCKAR, and CCK2R, known as CCKBR. CCK is a pre-pro-hormone synthesized by enteroendocrine intestinal cells, by I cells of the duodenum and jejunum, and by G cells of the stomach. Proteolytic cleavage of CCK generates a broad range of bio-active peptides among which are CCK-4, CCK-8 and CCK-8S (sulfated) (see review [6]). In 1975, the discovery of CCK-4 and CCK-8 peptides in the nervous system [7,8], prompted the characterization of CCK as a neuropeptide with pro-nociceptive and neurotransmitter properties. Pro-nociceptive effects are mainly mediated by the activation of CCK2R. CCK-4, CCK-8 and their two receptors, CCK1R and CCK2R, which are expressed in areas of the brain associated with pain modulation and other processes such as memory, anxiety and thermoregulation. More recent studies have confirmed that the CCK system interacts with numerous neuropeptides involved in pain regulation (dopamine, glutamate, opioid, etc.) [9,10,11], and acts as a neuromodulator in sensation and pain tracts via CCK2R. The established role of CCK in directly facilitating nociception, has led to the development of several CCK2R antagonists which have been shown to successfully alleviate pain in rodent models [12,13]. Extensive research into non-peptide, small-molecule CCK2R antagonists has identified numerous promising lead compounds and clinical candidates, yet none has thus far made it into the clinic due to their unfavorable, ineffective and variable biological effects observed in clinical trials. This review summarizes the current knowledge supporting a role for CCK2R and its modulators for the treatment of pain. More specifically, we focus on the cellular localization of CCK2R in the sensory nervous system and discuss in further detail the molecular mechanisms and signal transduction pathways involved in controlling pain perception. We then provide a comprehensive overview of the most successful compounds targeting CCK2R and report recent advances in pharmacological strategies used to achieve CCK2R modulation. Lastly, the biological and clinical relevance of CCK2R as a promising target for the development of new treatments for pain management is emphasized.

## 2. CCK2R Structure and Functions

### 2.1. Gene Localization and Related Diseases

Human CCK2R was cloned for the first time in 1993 [14]. *Cck2r*, the gene coding for CCK2R, which is located on the terminal short arm of chromosomes 11 in humans (11p15.4), is 12 kbp in length and is composed of 5 exons. According to the Ensembl database, three transcripts have been identified in humans. CCK2R splice variants and mutations have been shown to be involved in cell proliferation and cancer pathogenesis [15]. Mutations, as well as overexpression of CCK2R, are associated with carcinogenesis via the modulation of processes including cell proliferation and cell adhesion.

### 2.2. Structural Features of the CCK2R

CCK1R and CCK2R belong to the family of G-protein-coupled receptors (GPCRs). The two genes share only 50% sequence homology, mostly in domains characteristic of GPCRs and in sequence signatures that are required for receptor activation, which might explain their differences in affinity for ligands [16]. Though there remain some controversies, CCK2R can be generally associated with Gq protein, and to a lesser extent with Gi, contrary to CCK1R which is associated with Gs protein [17]. The activated Gq pathway passes through phospholipase C beta (PLCβ), which hydrolyzes phosphatidylinositol-4,5-bisphosphate (PIP2) to inositol-trisphosphate (IP3), leading to the release of intracellular Ca^2+^ stores into the cytoplasm, and to diacylglycerol (DAG), leading to the activation of protein kinase C (PKC). Ca^2+^ is a major effector of neuronal function, involved in neurotransmitter release and membrane excitability. It has been shown that PKC can play a crucial role in the regulation of GPCRs [18]. GPCRs are comprised of seven α-helical transmembrane domains, an extracellular *N* terminus with three extracellular loops, and an intracellular C terminus with three intracellular loops. GPCRs are a major class of proteins that are potentially involved in pain transmission and so, represent a preferred therapeutic target for novels analgesics [19]. For example, serotonin, apelin, dopamine, GABA_B_, opioid receptors and the recently clinically-implicated angiotensin II type 2 receptor (AT2R), all use signaling that passes through the GPCR family proteins and have roles in pain modulation [19,20].

### 2.3. Cellular Localization and Tissue Distribution in the Nervous System

CCK2R is localized in the plasma membrane and can be internalized for desensitization, an essential mechanism for maintaining physiologically appropriate cellular responses to extracellular stimuli. The internalization of the receptor can be decreased by removal of the C terminus, without affecting G protein coupling [21].

Only a few studies have investigated the expression of CCKRs in the human brain. Most of these explored the binding of radiolabeled ligands, which did not allow the discrimination between CCK1R and CCK2R [22,23]. However, a study using integrative data from several sources (transcriptomics, single-cell genomics, in situ hybridization and antibody-based protein profiling) has shown the localization and expression level of the receptors in human, pig and mouse brain [24]. This database detected CCK2R mRNA in most parts of the human brain, except the thalamus. Some of the structures that express CCK2R are directly implicated in the modulation of pain, such as the hypothalamus, the basal ganglia and the amygdala.

**Figure 1:**
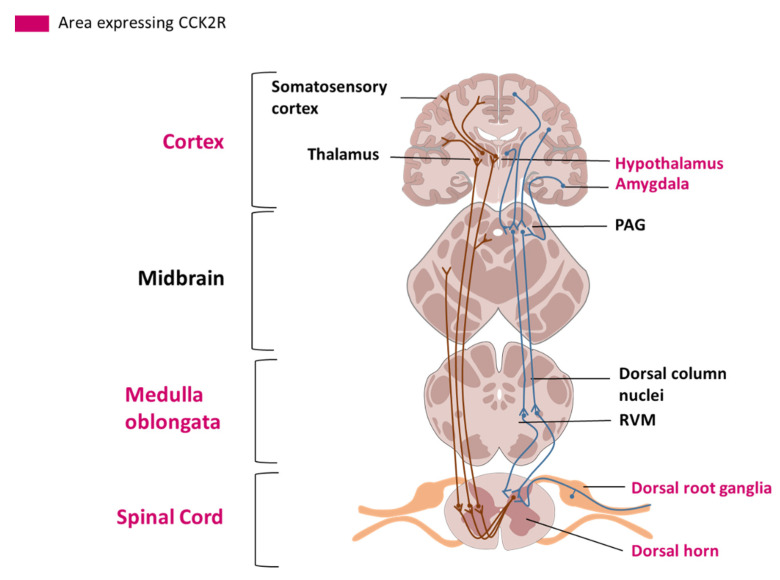
**Representation of the spinothalamic tract and descending pain modulation pathway.** Areas where CCK2R has been detected are labelled in pink. A: Ascending pain pathways. Nociceptors (C and Aδ fibers) send pain information from peripheral tissues, their cell bodies are localized in the dorsal root ganglia (DRG). Fibers transmit nociceptive signals to a second order neuron in the dorsal horn of the spinal cord. The second order neuron then crosses over to the opposite side, where it forms the ascending spinothalamic tract. This tract takes signals to nuclei in the medulla oblongata and midbrain on the way up to the thalamus. The thalamus relays the information to the somatosensory and insular cortex, as well as cortical regions mediating different aspects of the pain experience such as affective responses in the cingulate cortex. B: Descending pain modulation pathways: Information from the environment and some motivational states can activate this top-down pathway. Several areas in the limbic forebrain including the anterior cingulate and insular cortex, nuclei in the amygdala and the hypothalamus, project to the midbrain periaqueductal grey (PAG), which then modulates ascending pain transmission from the afferent pain system indirectly through the rostral ventromedial medulla (RVM) in the brainstem.

The hypothalamus has a role in modulating the affective dimension of pain and is part of the spinothalamic tract, the principal pain tract [25] (Figure 1). The basal ganglia (mainly including the putamen and the caudate nucleus, and the nucleus accumbens (NAc)) have been principally studied for their motor functions, but are also involved in different aspects of pain including the sensory-discriminative, affective and cognitive dimension of pain and the modulation of nociceptive stimulus intensity information [26]. The amygdala plays an important role in emotional responses and affective states, in disorders such as learned fear, anxiety and depression, and also in pain modulation [27].

Low level expression of CCK2R has been detected in the spinal cord [24]. Interestingly, the expression of CCK2R in the human peripheral nervous system has not been explored. In rats, CCK2R mRNA has been detected in dorsal root ganglia (DRGs) using in situ hybridization. Only 3% to 4% of all DRG neurons in physiological conditions expressed CCK2R mRNA [28]. Another study using [^125^I]-CCK-8S labelled with Bolton-Hunter reagent to localize its binding site in DRG, led to the same conclusion [29]. Moreover, in a model of unilateral peripheral transection of the sciatic nerve in the rat [28], in situ hybridization highlighted an increase in CCK2R mRNA in the L4 and L5 DRGs after axotomy. Localization of CCK2R in nociception-linked areas and upregulation of CCK2R mRNA after traumatic lesions underlined the interest of evaluating the roles of CCK2R in pain modulation.

## 3. Involvement of CCK2R in Pain Modulation

**Figure 2:**
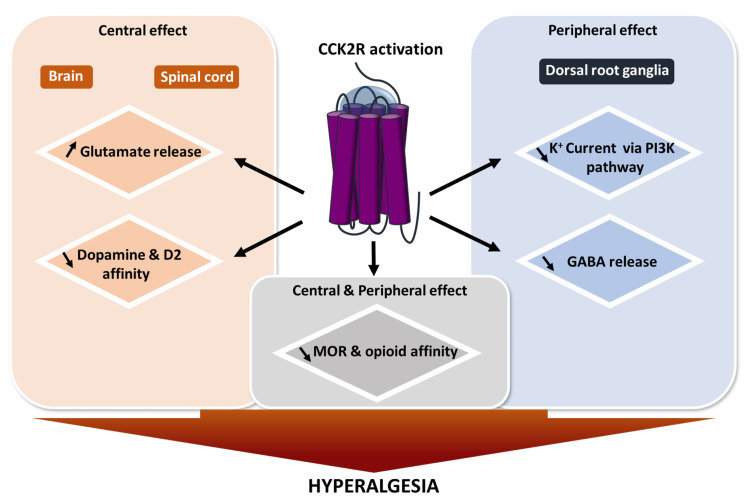
**Effect of CCK2R activation on pain modulation.** Summary of known CCK2R actions after activation. Most of its interactions induce a mechanism leading to hyperalgesia. GABA: gamma-aminobutyric acid; D2: dopamine receptor 2; MOR: µ opioid receptor; PI3K pathway: Phosphoinositide 3-kinase pathway.

Pain is defined by The International Association for the Study of Pain (IASP) as “an unpleasant sensory and emotional experience associated with actual or potential tissue damage, or described in terms of such damage”. Neuropathic pain is defined as pain caused by a lesion or a disease affecting the somatosensory nervous system. The development of neuropathic pain arises from multiple and complex pathophysiological mechanisms making its evaluation and management difficult. Hence, gaining knowledge about pain-underpinning mechanisms and their translation into symptoms might eventually allow a more appropriate therapeutic approach to treat each patient (Figure 2).

### 3.1. The Heterodimerization of CCK2R and the Opioid Receptor Reduces Opioid Efficacy

Since 1983, the role of CCK in hyperalgesia has been studied, in particular its interaction with opioid receptors [30]. Opioids are the most powerful analgesics currently used for the treatment of pain, yet repeated administration induces tolerance which significantly decreases their analgesic effect. Opioids bind to opioid receptors belonging to the GPCR family, namely μ-opioid receptor (MOR), δ-opioid receptor (DOR), and κ-opioid receptor (KOR). Those receptors are localized in primary sensory neurons, the spinal cord, brainstem, midbrain and cortex. Binding to the opioid receptor induces a decrease in neuronal activity and an inhibition in the release of pro-nociceptive neurotransmitters [31].

Many studies have shown that CCK-8, when delivered intrathecally, via intracerebral injection or systemically, reduces the effect of opioids [32,33]. CCK2R likely contributes to this anti-analgesic effect. In rats, the selective blockade of CCK2R using the antagonist L365,260 enhanced morphine analgesia and this effect is 40 times more potent than that obtained with the CCK1R antagonist, L365,031 [34]. More recently, a study has demonstrated direct heterodimerization of MOR/CCK2R consecutively to CCK-8 binding. The authors identified the third transmembrane domain of MOR as being required for the interaction with CCK2R. This interaction is the basis of the inhibition of MOR signal transduction and the antagonism of morphine analgesia [11]. Such data strengthen a rationale for disrupting the MOR/CCK2R interaction in order to enhance opioid analgesia.

### 3.2. CCK/CCK2R Facilitates Glutamate Release

It is well-established that CCK is highly expressed in the hippocampus [35]. Studies have shown that glutamate and CCK-8 are co-localized in the cortico-striatal pathway [36]. A study using whole-cell recordings from rat hippocampal slices, showed that CCK binding to CCK2R substantially increased releasable glutamate vesicles in the hippocampus [37].

Glutamate is the most abundant excitatory neurotransmitter in the brain, and considering its pivotal role in pain sensation and transmission, modulation of the glutamate receptor activity represents a promising potential target for pain management. As an example, the use of NMDA or AMPA glutamate receptor antagonists favorably reduce thermal and mechanical pain thresholds in both a model of inflammation and also in normal rats [9,38]. Thus, by facilitating the release of glutamate, CCK/CCK2R interaction promotes hyperexcitability in the hippocampus and likely an increase in nociceptive pain. Therefore, inhibition of the CCK2R could have an analgesic effect other than that mediated by opioid receptors.

### 3.3. CCK/CCK2R Increases Dopamine

A study using an in vitro filter wipe-off technique, revealed a modulatory role of CCK-8 on dopamine release in the rat NAc [39]. Specifically, activation of CCK2R by CCK-8 reduced the affinity of the D2-like receptor in the brain. This observation was confirmed by microdialysis of CCK-8 in rat NAc, showing an increase in dopamine release.

Dopamine has a central role in pain modulation [40], and the disruption of dopamine homeostasis in the central nervous system could contribute to pain chronicity. Dopamine actions are mediated by two families of receptors: the D1-like family receptors and the D2-like family receptors, which, respectively, activate and inhibit the release of dopamine. Stimulation of the D2-like receptors has an analgesic capacity. This effect has been shown for both inflammatory pain and in thermal nociception [41,42]. Moreover, the use of D2 antagonists has demonstrated a pro-nociceptive effect [41].

As outlined above, CCK2R activation mediates the release of dopamine, an excitatory neuromodulator, while CCK2R decreases the affinity of dopamine for its receptor D2. This mechanism could lead to pro-nociceptive effects via hyperexcitability.

### 3.4. CCK/CCK2R Decreases GABA Release

Pre-incubation of rat DRG neurons with CCK-8S prior to application of gamma-aminobutyric acid (GABA) resulted in attenuation of GABA-induced depolarization [43]. This effect was reversed by Ly225,910, a CCK2R antagonist. The CCK-8S-induced inhibition of GABA-evoked membrane potential can also be suppressed with a PLC inhibitor. The authors hypothesized that binding of CCK-8S to CCK2R would activate PKC, which would then phosphorylate the GABAA receptor inhibiting its function in DRG neurons. By this mechanism, the pre-synaptic inhibitory role of GABA would be disinhibited leading to induction of nociception activity in the spinal cord [43].

GABA also has a significant role in pain modulation, which is suggested by the anatomical distribution of GABA receptors (GABAA and GABAB) and evidence of the action of GABA agonists on the nociceptive response. GABA receptors are present in the spinal cord and brain areas that are associated with mediating and perceiving pain [44,45,46,47]. Moreover, GABA receptors have been localized on primary afferent A-δ and C fibers, which conduct mechanical and thermal nociceptive information.

### 3.5. CCK/CCK2R Decreases K^+^ Channel Currents

Inhibition of the transient type-A K^+^ current by CCK-8/CCK2R activation has been recently shown in small-sized DRG neuron extracts from mice [48]. DRG neurons express a variety of A-type K^+^ channels that regulate membrane excitability. In DRG neurons, a decrease in A-type K^+^ current induces an increase in neuronal excitability and is, thus, associated with chronic pain [49]. The underlying mechanism of CCK2R-mediated modulation of A-type K^+^ currents involves the PI3K/SRC/JNK pathway. This signaling cascade results in the phosphorylation and, thus, the inhibition of A-type K^+^ channels [48]. In the same study, the authors showed that intraplantar administration of CCK8 affected nociception in mice, characterized by mechanical and heat hypersensitivity, and that this effect was mediated by CCK2R and modulation of A-type currents.

## 4. Development of Pharmacological Modulators

The search for and development of selective CCK2R agonists or antagonists has been a major challenge since the 1990s with potential applications spanning various medical areas. The sites and mechanisms of binding for endogenous and non-peptide ligands of CCK2R have been studied in detail, which has provided the knowledge for understanding the molecular basis of these interactions [50]. A description of ligand-binding sites in the CCK2R, defined by experimental and computational methods, has been reported elsewhere [51]. Here, we summarize the most potent and selective antagonists and, additionally, the endogenous agonists of CCK2R (Table 1).

## 5. Pharmacological Modulation of CCK2R in Preclinical Models of Pain

Numerous rodent models of pain have been developed and characterized in recent years. Each model is representative of one of three types of pain: neuropathic, inflammatory or nociceptive. Different mechanisms are involved according to the cause of pain. Neuropathic pain is caused by a lesion or disease of the somatosensory system, including peripheral fibers (Aβ, Aδ and C fibers) and central neurons. Neuropathic pain can be central or peripheral, and results from trauma (spinal cord injury, stroke, post herpetic neuralgia) or toxic injury (diabetes, chemotherapy). Inflammatory pain is caused by the release of chemicals that sensitize nerve terminals at the site of inflammation (for example, rheumatoid arthritis, inflammatory bowel disease). Nociceptive pain results from normal noxious stimuli, without alteration of nerve fibers or endogenous chemical events (Table 2).

### 5.1. Model of Traumatic Injury at the Central Nervous System Level

The role of CCK/CCK2R in central neuropathic pain was investigated in a rat model of spinal cord injury (SCI) [13]. Following injury, animals developed mechanical allodynia, which was reduced by systemic or intrathecal administration of the CCK2R antagonist, CI-988 (see Table 1). Moreover, CCK mRNA expression was increased on the ipsilateral side at the spinal segments caudal to the SCI. These results suggest that the upregulation of spinal CCK may contribute to the development or maintenance of central neuropathic pain after SCI, via CCK2R activation [13]. Therefore, compounds similar to CI-988 may be useful for central pain management caused by traumatic injury.

### 5.2. Model of Traumatic Injury at the Peripheral Nervous System Level

Traumatic injuries to the peripheral nerves can be attributed to a combination of mechanisms ranging from simple traction or stretch to ischemia, burns and electrical injuries. In general, traumatic peripheral nerve injuries impede the recovery of functions, return to work and quality of life.

Following chronic constriction injury (CCI) of the sciatic nerve, wild type (WT) mice displayed chronic neuropathic pain symptoms from the second postoperative day to the end of the tests (44 days), whereas CCK2R^-/-^ mice did not display chronic hyperalgesia [60]. This study suggests the involvement of CCK2R in the establishment of mechanisms leading to neuropathic pain following traumatic nerve injury.

### 5.3. Model of Burn Pain

In a model of hind paw burn injury in mice, the involvement of CCK2R in pain [61] was also supported. Burn-induced pain is caused by damage to peripheral nerve endings (neuropathic pain) and inflammatory processes (inflammatory pain). The burn injury model was described from both transcriptomic and pharmacological perspectives. The transcriptomic analysis of DRG neurons showed that the *Cck2r* gene was upregulated after induction of the burn injury. Several behavioral tests were performed to assess the impact of the burn (motor assessment, paw thickness, gait analysis, Hargreaves test, electronic von Frey test), the efficacy of opioid (meloxicam, amitriptyline, gabapentin and oxycodone) and the effect of the CCK inhibitor proglumide on pain. Opioid drugs, injected intraperitoneally (i.p.) once daily during the experiment, partially reversed mechanical allodynia but had no effect on thermal allodynia and gait abnormalities induced by burn injury. Proglumide (30 mg/kg/day, i.p.), significantly alleviated mechanical allodynia alone or in combination with oxycodone at low dose (1 mg/kg, i.p.). Oxycodone alone at 1 mg/kg did not produce any analgesia. Co-administration of proglumide with low-dose oxycodone appeared to improve paw withdrawal force but was not statistically significant; however, it did alleviate thermal allodynia. CCK2R antagonists may be useful clinically as adjuvants to decrease opioid requirements and improve analgesic management.

In this study, the only CCK2R antagonist tested was proglumide, which is not a specific antagonist of CCK2R. It may have a direct interaction with δ opioid receptors, which can mask a potential effect of CCK2R blockade.

### 5.4. Model of Diabetic Neuropathy

Diabetic neuropathy most often affects nerves in the legs and feet. Depending on the affected nerves, painful diabetic neuropathy symptoms can range from burning, sharp or aching pain and numbness in the legs and feet, to problems of the digestive system, urinary tract, blood vessels and heart.

The role of spinal CCK2R in thermal allodynia and hyperalgesia was investigated in a mouse model of streptozotocin-induced diabetic neuropathy [12]. Diabetic mice displayed thermal hyperalgesia and allodynia characterized by a reduced time response to the tail flick in response to a heat test. In diabetic mice, blockade of the spinal CCK2R by intrathecal (i.t.) CI-988 injection showed a significant increase in tail response latency compared with untreated diabetic mice. Moreover, CCK2R activation by CCK-8 i.t. injection in non-diabetic mice reduced the latency of the tail response, and this effect was reversed by CI-988 pretreatment. As observed with CCK-8, the injection of a protein kinase C (PKC) activator reduced tail response latency and was also reversed by CI-988. This observation suggested that the activation of CCK2R could be involved in thermal hypersensitivity observed in diabetic neuropathy, via activation of the PKC pathway. Blockade of spinal CCK2R could, therefore, be a therapeutic strategy to alleviate some pain associated with diabetes [12].

## 6. Clinical Relevance of CCK2R Pharmacological Targeting

### 6.1. Placebo

The placebo effect describes the beneficial psycho-physiological result observed after the administration of a substance or the performance of a therapeutic act, regardless of the expected intrinsic efficacy of the treatment. The placebo effect seems to be mediated by activation of μ-opioid receptors [62].

The interaction already highlighted between CCK2R and opioid receptors demonstrates the role of CCK2R in the placebo response [63]. The use of pentagastrin, as a CCK2R agonist, disrupted the placebo effect in human subjects. In a double-blind study 40 volunteers were divided into 4 groups: natural history (control) group, hidden pentagastrin group, placebo group and placebo+ pentagastrin group [63]. In the three treatment groups, morphine was injected intravenously 15 min before inducing pain with a tourniquet on three non-consecutive days. On day 4, morphine was replaced by a placebo (saline solution) and/or pentagastrin. In the group receiving placebo only, similar pain was observed compared to the response with morphine, whereas in the groups receiving pentagastrin, the pain was at the level of control (without morphine). These results showed that opioid-induced placebo responses were abolished by the administration of pentagastrin. There would appear to be a balance between opioid receptors and CCK2R.

### 6.2. Postoperative Pain

Postoperative pain is defined as acute pain experienced by the patient after a surgical procedure. Good management of postoperative pain is important in reducing complications and facilitating rehabilitation. Postoperative pain relief is mainly based on opioids, and so is associated with side effects such as constipation, respiratory distress and the development of tolerance to administered opioids.

In 1988, a clinical trial assessed the interactions between morphine and proglumide in 80 patients [64]. All patients suffered from moderate to severe postoperative pain following abdominal or gynecological surgery. They were divided into four groups that all received morphine on demand at 3 mg via the i.v. route: alone; with 0.05 mg proglumide (i.v.); with 0.5 mg proglumide; with proglumide at 50 mg. Morphine consumption, pain scores and side effects were evaluated 8 h after the first administration of morphine. At the end of this clinical trial, there was no difference between the groups either for drug consumption, pain scores or side effects. It was therefore concluded that proglumide does not potentiate morphine analgesia in a clinical postoperative setting and does not modify side effects [64].

The use of proglumide has also been evaluated in 60 patients with dental postoperative pain [65]. Proglumide administered at 0.05 mg (i.v.) in combination with morphine at 4 mg (i.v.) achieved a significantly greater analgesic effect than morphine at 4 mg or 8 mg alone, with no evidence of increased side effects. Proglumide alone had no effect even at higher doses (0.5 and 5 mg) [65].

These two studies, evaluating the efficacy of proglumide in postoperative pain, gave contradictory results, which could be explained by the use of different pain scales. Indeed, visual analogue scales (VAS) are more sensitive than category scale measurements [66]. Moreover, it would appear that the potentiation of morphine analgesia by proglumide, follows a biphasic dose-response, with low doses being more effective than high.

### 6.3. Cancer Pain

Cancer pain may have multiple forms. Pain can be directly caused by the tumor itself, as it grows into or destroys adjacent tissue. As a tumor grows, it can press on nerves, resulting in neuropathic pain. The tumor can also release chemicals that can cause inflammatory pain, and cancer treatments, including surgery, radiation and chemotherapy, can also cause pain.

Proglumide has been tested in cancer pain in humans [67]. A double-blind crossover study was carried out on 43 patients suffering from metastatic cancer pain. Patients received in one arm, a full analgesic dose of morphine plus placebo i.v., and in the other arm, half the analgesic dose plus 50 mg of proglumide. Pain was analyzed using 8 VAS and 3 Tursky verbal rating scales (TVS). Analgesia was similar in the two arms, suggesting that proglumide potentiated the effect of morphine [67].

### 6.4. Neuropathic Pain

The analgesic effect of the CCK receptor antagonist L365,260 on chronic human neuropathic pain that is unresponsive to current treatment, has been studied in a randomized, double-blind, placebo-controlled crossover study [68]. Forty adult subjects taking morphine for neuropathic pain were enrolled in this assay. Each subject received three treatments in varying order, separated by a washout period of between 5 and 14 days. Treatments consisted of a dose of morphine with placebo, 30 mg of L365,260 taken orally or 120 mg of L365,260, each treatment administered for 2 weeks. In contrast to preclinical studies, L365,260 failed to show an antalgic effect [68].

## 7. Conclusions/Critical Perspectives

Many compounds acting on CCK2R have shown promising results in preclinical models of pain. However, most of these compounds did not produce the desired effects in clinical trials. The discordance of results between preclinical and clinical studies may be explained in part by the differences in expression and distribution of CCK2R between rodents and humans. Indeed, despite a gene sequence homology of 90–97% across species [2], the location of CCK2R in the nervous system is not the same. According to Sjöstedt et al., the pig would be a better model for the study of the human brain [24], particularly as CCK2R distribution in the human brain is closer to that in the pig than the mouse. However, it should be noted that CCK2R is expressed in the thalamus in both mice and pigs, but is absent in humans [24]. The thalamus is an important structure mediating pain transmission, so this may contribute to interspecies variation in the efficacy of CCK2R antagonists. Moreover, inconsistency between assessment tools used in rodent models and clinical endpoints in human studies may also be a technical contributor to differences observed between species. In fact, most studies to evaluate drug efficacy in rodent models use very restricted nociceptive endpoints (allodynia, hyperalgesia) measured by behavioral tests, whereas neuropathic pain symptoms are wider and more complex, including paresthesia or dysesthesia, than these endpoints can detect. In addition, primary endpoints in clinical trials vary, adding another degree of complexity to the translation of preclinical data into clinical efficacy. Finally, the effective dose of CCK2R antagonists seems to be restricted and, regardless of whether dosing is assessed in clinical or preclinical studies, effective doses are often at the lower end [12,65]. Thus, the relationship between ligand and receptors does not seem to be linear.

Data are scarce concerning the exact localization of CCK2R in the nervous system, especially in the peripheral nervous system, thus limiting our understanding of CCK2R’s mechanism of action. In rodents, some information on CCK2R expression in DRG indicates a very low expression in neurons [28]. However, DRG are not only composed of neurons but also satellite glial cells, which are crucial for proper functioning of the sensory nervous system [69], immune cells and endothelial cells. Satellite glial cells could be involved in pain processes and also in immunity. In addition, studies have identified the expression of CCK2R on different immune cell types, such as macrophages and T lymphocytes [70,71]. Further studies are needed to accurately define the localization of CCK2R, particularly in the sensory nervous system to help elucidate its role and that of the various cell populations in pain modulation.

The lack of positive results of CCK2R clinical studies on pain management, may be due to the poor bioavailability and efficacy of administered agents [72]. Some of the antagonists discovered early on are in fact partial agonists (L365,260) or inverse agonists, which calls for a reconsideration of the use of these compounds and the need for extensive characterization of newer compounds [73]. The lack of a precise 3D structure of CCK2R and limited knowledge of the essential structural features of its non-peptide ligand-binding site(s) hamper the development of CCK2R antagonists [73]. Most CCK2R antagonists have been studied in anxiety, fear and memory [10,74,75].

The value of CCK2R antagonist-based therapies lies in the complex and multiple signaling activated by the CCK2R. As mentioned above, CCK2R can interact with several other GPCRs and, thus, modulate the interaction with their ligands, shifting the discharge of neurotransmitters or ion transport. These varied interactions greatly complicate understanding of the mechanism of action of CCK2R that seems to differ depending on which part of the nervous system is involved.

Despite gaps in our understanding of the role of CCK2R in pain modulation, an increasing number of transcriptomic analyses underway in pain models, are highlighting the upregulation of CCK2R in several models including abdominal pain in patients with irritable bowel syndrome [76], a model of lipopolysaccharide-induced lung inflammation [77] and a model of deletion of the NMDA receptor inducing hypersensitivity [78]. This upregulation demonstrated a central role of CCK2R in pain development.

To conclude, there is accumulating evidence that CCK2R plays a crucial role in the modulation of pain and that its blockade is a valid therapeutic strategy in the management of pain. Further preclinical investigations with more specific antagonists, appropriate dosing and models are needed to understand the mechanism(s) of action of CCK2R. Several promising new CCK2R antagonists have already been administered in human clinical trials with good safety profiles. Thus, these compounds represent new opportunities in analgesia pharmacology.

## Figures and Tables

**Table 1:** Main CCK2R Agonists and Antagonists.

Agonists			Target	Antagonists		
	Affinity	Ref		Synthetic	Affinity	Ref
CCK-4 (endogenous)	Ki = 0.11 µM	[52]	CCK2R	CI-988 (PD-134 308)	Ki = 0.5 nM	[53]
				L365,260 (benzodiazepine analogue)	Ki = 7 nM	[54]
CCK-8 (endogenous)	Ki = 0.3 nM	[14]		Ly225,910	Ki = 0.2 nM	[55]
Pentagastrin (synthetic)	Ki = 0.0029 µM	[52]		Netazepide (YF476)	Ki = 0.19 nM	[56]
				YM-022	Ki = 68 pM	[52]
CCK-8S (endogenous)	CCK1R Ki = 1.41 nM	[57]	CCK1R & CCK2R	Proglumide	CCK1R: IC50 = 6 µM	[58]
	CCK2R Ki = 0.34 nM				CCK2R: IC50 = 11 µM	

This table summarizes the endogenous CCK2R agonists and their affinity, and provides a non-exhaustive list of the most potent and selective CCK2R antagonists. Ki: dissociation constant; IC50: the half minimal inhibitory concentration.

**Table 2:** Preclinical studies highlighting the role of CCK2R in pain.

Model Type	Species	Injury	Treatment	Effect of CCK2R Modulation	Refs
**Central nervous system**					
Traumatic model	Male Sprague–Dawley Rats	Hemisection at T13 on left side	CI-988 (CCK2R antagonist) (i.t.) and systemic injection	CI-988 reduced allodynia	[13]
	129S4 CCK2R^-/-^ mice	Chronic constriction injury	No treatment	In midbrain and medulla, CCK2R^-/-^ mice showed reduced expression of MAPK pathway and cytokine production, compared to WT mice	[59]
**Peripheral nervous system**					
Traumatic model	129sv⁄C57BL6 CCK2R^-/-^ mice		Naxolone (opioid receptor antagonist) injection (i.p.)	CCK2R^-/-^ mice showed reduced sensitivity without treatment, naxolone injection increased mechanical allodynia, compared to WT Mice	[60]
			L365,260 (i.p.)	High dose of L365,260 decreased the sensitivity of WT mice	[60]
		Ligation of the sciatic nerve		CCK2R^-/-^ mice did not display hyperalgesia, and exhibited less inflammation in sciatic nerve	[60]
	Rats	Unilateral peripheral transection of sciatic nerve		CCK2R was overexpressed in ipsilateral side of axotomy	[28]
	129sv⁄C57BL6 CCK2R^-/-^ mice	Chronic constriction injury		CCK2R^-/-^ mice had less microglial infiltration in nerve compared to WT	[60]
Burn model	C57BL6 mice	mild burn injury	Proglumide (30 mg/kg, i.p.), meloxicam (5 mg/kg, i.p.), gabapentin (100 mg/kg, i.p.), oxycodone (10 mg/kg, i.p.)	Proglumide decreased mechanical allodynia alone or with co-administration of oxycodone at low dose (1mg/kg, i.p.) and alleviated thermal allodynia	[61]
Diabetic neuropathy model	ICR mice	Diabetic mice (injection of streptozotocin)	CI-988 (i.t.)	CI-988 increased latency of tail response in diabetic mice	[12]
			CCK-8 (i.t.)	CCK-8 injection decreased latency of tail response in WT mice, reversed by pretreatment with CI-998	[12]

i.p.: intraperitoneal; i.t.: intrathecal.

## Data Availability

Data sharing is not applicable to this article.
